# Structure–Function Correlation in Switchable DTE@MOF Hybrids: Tracking Local Dynamics and Fatigue Pathways

**DOI:** 10.1021/acs.jpcc.5c06348

**Published:** 2025-12-02

**Authors:** Markus Rödl, Eva Neuner, Armin Penz, Axel Gansmüller, Thomas S. Hofer, Heidi A. Reichl

**Affiliations:** † Institute of General, Inorganic and Theoretical Chemistry, 27255 Universität Innsbruck, Innrain 80-82, Innsbruck 6020, Austria; ‡ CNRS, CRM2 UMR 7036, Université de Lorraine, Boulevard des Aiguillettes, BP 70239, Vandoeuvre les Nancy 54506, France

## Abstract

The origin of optical properties of non-covalently inserted photoswitchable molecules inside porous metal–organic frameworks (MOFs) remains a challenge due to the high mobility and spatial freedom of the embedded dye. Both before and after switching, dye-MOF interaction motifs are subject to changes, which might significantly impact the resulting photochromic response with respect to fatigue resistance. Within this work, the light-induced isomerization processes of a P-type chromophore (dithienylethene) inside the robust UiO-67 are studied with respect to structural rearrangements. For the first time, solid-state NMR (ssNMR) data were utilized next to X-ray diffraction experiments to characterize the embedment and physical state of the photoswitchable dye by tracing changes in both the MOF and guest signature. Moreover, diffuse reflectance spectroscopy measurements revealed slight fatigue originating from a unique nanoconfinement effect within the MOF pore. The open photoinactive form is preferably present in an MOF side pore, causing fatigue of the photoswitching. Molecular dynamics (MD) simulations at the semiempirical quantum chemical level of theory provided the atomic perspective to understand the underlying host–guest interaction, involving the occupation of different pores of UiO-67 by the open and closed form of the embedded chromophore and the tremendous stabilization of the compact photoinactive conformer.

## Graphical abstract

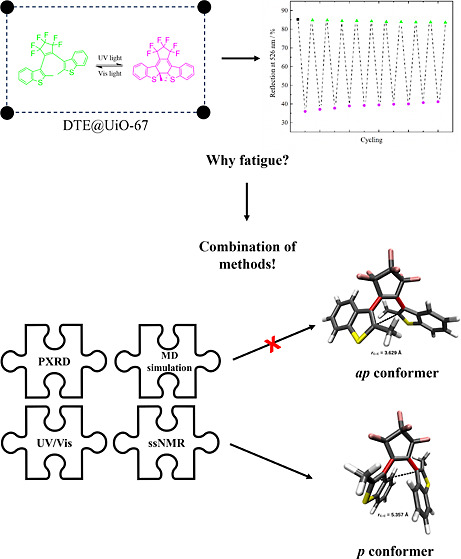

## Introduction

Understanding the insertion processes and corresponding behavior of photochromic dyes inside metal–organic frameworks (MOFs) has become a widely studied research field over the past few years. Photochromic dyes are species, which reversibly transform between two or more conformers upon irradiation with light of a specific wavelength.^1^ These light-induced isomerization processes are mostly present in the dissolved state, but due to spatial restrictions, photochromism is rarely observed in the solid state, which is why the insertion of such dyes into porous materials such as MOFs has become an attractive way to overcome this problem. MOFs are crystalline, porous materials consisting of inorganic and organic compounds, which form 2D or 3D networks containing potential voids.^2^ Robson,^3,4^ Yaghi,^5,6^ and Kitagawa^7^ were recently rewarded with the Nobel Prize for their discovery and development of this outstanding class of materials. MOFs represent ideal host matrices for the embedment of photochromic dyes as noncovalently attached guest molecules in an extrinsic approach, leading to so-called switch@MOF hybrid materials.^7–15^ As the dye molecules are mobile and separated from each other inside the MOF pores, these frameworks enable photoswitching in the solid state. Notably, the switching unit can also be integrated as part of the framework itself, which is referred to as intrinsic introduction.^16^ More details on recent advances and developments in photochromic MOF chemistry can be found in these reviews.^17–20^ Switch@MOF composites offer a wide range of potential applications, also thanks to the fact that the MOFs are variably designable, and as a result, the dye properties can be effectively directed and influenced by the given MOF confinement. In fact, apart from the application in memory and data storage devices,^21,22^ they can also be used in various sensor materials,^23^ conductive materials,^11,24,25^ or for remote-controllable membrane separation.^26–28^ For technological purposes, photochemically stable switches are of great interest to study the host-dependent enclosure characteristics. Diarylethenes and dithienylethenes^29^ represent one of such compound classes, exhibiting high fatigue resistance and further being solely switchable by light (P-type chromophore).^30,31^ Their photochromic response has been studied in a dissolved state, but also when being embedded in MOFs.^32,33^ Following the insertion process and investigating the optical response from a structural point of view remains a challenging task, as, e.g., conventional methods such as powder X-ray diffraction (PXRD) cannot exclude dye adsorption on the MOF surface. A comprehensive understanding of the system can be achieved by analyzing host- and guest-specific modulations through diffraction and spectroscopic techniques. Furthermore, comparing experimental data with theoretical models, in particular molecular dynamics (MD) simulations, enables the elucidation of optical properties from a structural perspective. To establish a chemically and thermally robust model system for this purpose, we chose the photochromic dye 1,2-bis­[2-methylbenzo­[*b*]­thiophen-3-yl]-3,3,4,4,5,5-hexafluoro-1-cyclopentene (DTE)^34^ and the MOF UiO-67 ^35,36^ as a suitable combination of a thermally stable photoswitch (pure P-type chromophore, solely switchable by light)^30,31^ and a thermally and chemically stable MOF host with large pores to simulate a liquid-like or gaseous environment for the DTE molecule. The respective structures and characteristics are depicted in Figure 1.

**Figure 1:**
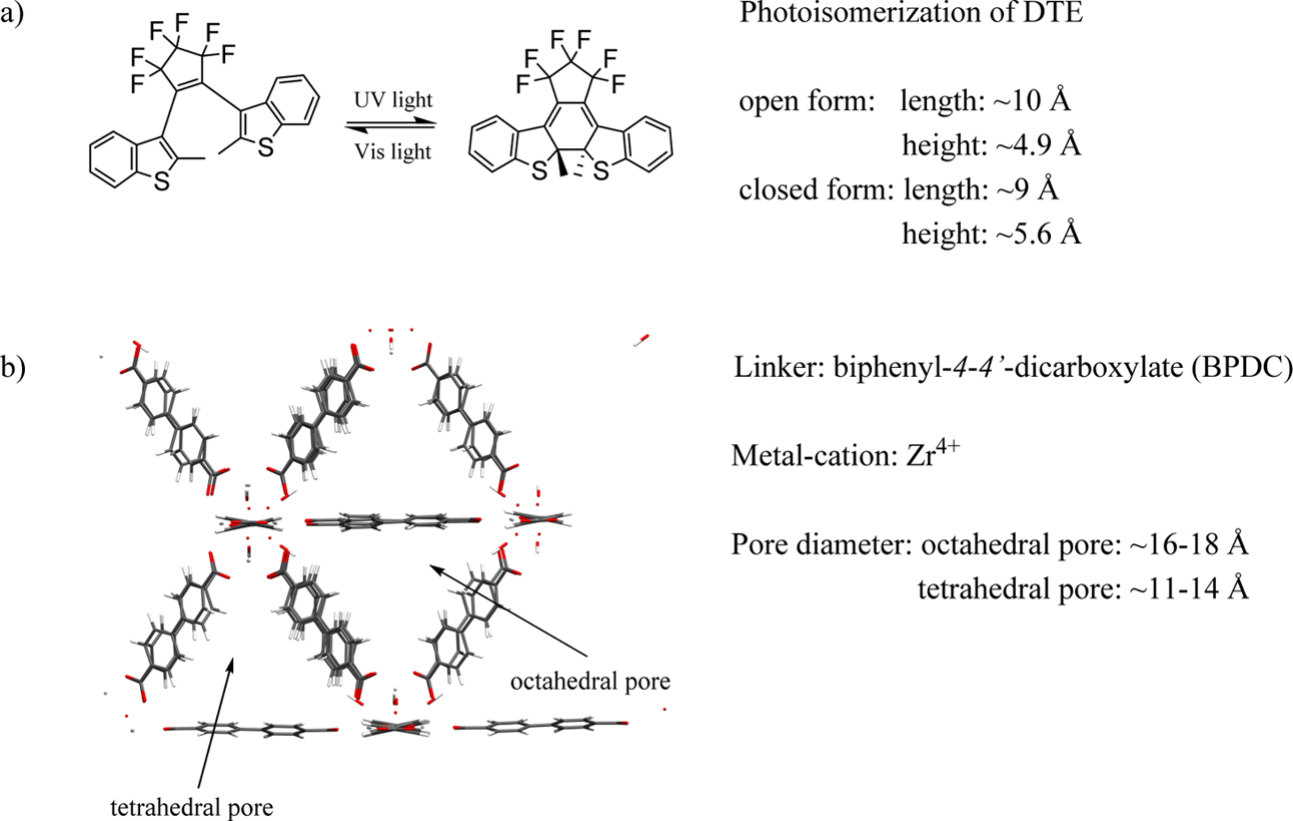
(a) Photoisomerization of DTE between the open colorless and the closed colored form upon irradiation with UV and visible light, estimated size of the open and closed isomer from a structural analogous DTE.^31^ (b) Structure of UiO-67 with specifications on linker, metal-cation, and pore diameters.^37,38^ The structural motif was visualized utilizing the VMD program package.^39^

The aim of this work was to (1) characterize the dye insertion by utilizing PXRD (for MOF-specific modulations upon insertion) and IR and ssNMR spectroscopy (for both MOF and dye-specific modulations upon insertion) and (2) to understand the optical properties as a function of structure. Here, the experimental studies of DTE@UiO-67 were complemented by extensive MD simulations. The latter have been employed in conjunction with the third-order density functional tight binding method DFTB3.^40^

## Experimental Section

### Chemicals

Commercially available ZrCl_4_ (Thermo Scientific), biphenyl-4–4′-dicarboxylic acid (H_2_BPDC) (Thermo Scientific), *N*,*N*′-dimethylformamide (DMF, Fisher Scientific), and 1,2-bis­[2-methylbenzo­[*b*]­thiophen-3-yl]-3,3,4,4,5,5-hexafluoro-1-cyclopentene (DTE) (TCI chemicals) were used without further purification.

### Synthesis of UiO-67

This synthesis was modified from Sannes et al.^35^ A mixture of ZrCl_4_ (2.3 g, 9.870 mmol), H_2_BPDC (2.4 g, 9.908 mmol), and DI H_2_O (0.25 mL, 14 mmol) was unified with 200 mL DMF, placed into a pressure-stable glass bomb and closed with a Teflon seal afterward. The system was heated to 95 °C and kept for 100 h in an oil bath. After being cooled to RT, the yellow-brown mixture was filtered off, leaving a yellowish solid. The received solid was washed with DMF three times and dried in air at RT overnight and then at 100 °C under air for 5 days and under reduced pressure for two days. To avoid the absorption of humidity, the powder was stored under an argon protective atmosphere. The phase purity was confirmed by PXRD (see Figure 1).

### Synthesis of DTE@UiO-67 Systems

DTE was incorporated into UiO-67 via a gas-phase loading process at 120 °C and a reduced pressure of ∼3×10^–3^ mmbar. In Table 1, the molar guest:host ratios and the weighed-in masses are listed.

**Table 1:** Weighed-in Masses and Temperatures for the Gas-Phase Synthesis of DTE@UiO-67^a^

guest/host ratio	*m*_guest_/mg	*m*_host_/mg
1:1	13.25	60

a Phase purity of the obtained hybrid system was confirmed by means of PXRD (see Figure 1).

### Powder X-ray Diffraction (PXRD)

To probe the phase purity of the MOF host, as well as the successful incorporation of the DTE, PXRD was applied. Measurements were carried out with a *Stoe Stadi P* diffractometer (Stoe, Darmstadt, Germany) in transmission geometry with Mo K_α1_-radiation (λ = 70.93 pm) utilizing a focusing Ge(111) primary beam monochromator and a Mythen 2 DCS4 detector. Data was collected in the 2θ range of 2.0–30.2° with a step size of 0.705°. Eight such scans were added to obtain the best signal-to-noise ratio. For each measurement, the respective sample was sealed in a glass capillary under an argon atmosphere to prevent absorption of humidity. All measurements were carried out at RT.

The Rietveld refinement was performed utilizing the Diffrac^plus^-Topas 4.2 software.^44,45^ The crystal structure solution of UiO-67 ^36^ was used as a starting point. The contribution of the diffractometer was adjusted by refining a LaB_6_ standard. The background was corrected with *Chebychev* polynomials to the 16th order.

### Liquid-State Nuclear Magnetic Resonance (NMR) Spectroscopy

^1^H NMR spectra were collected on a 400 MHz Bruker AVANCE 4 Neo spectrometer. Measurements were performed at room temperature and processed with MestReNova 9.0.1–13254. For each measurement, the samples were dissolved in 0.5 mL of DMSO and 25 μL of D_2_SO_4_ to decompose the host framework.^13^ ^1^H NMR spectra of both the individual components as well as the DTE@UiO-67 systems are shown in Figures S1 to S3, details on the composition determination can be found in Table S1, Supporting Information. For irradiation experiments, a Prizmatix PRI FC5-LED-WL (five high-power Fiberglas-coupled LED output with a potentiometer for manual power control) was used. Here, the same measurement conditions were chosen, but the sample powder was exposed to UV light (λ = 310 nm) for 10 min before digesting the sample and transferring it to the NMR spectrometer in the dark.

### Solid-State Nuclear Magnetic Resonance (NMR) Spectroscopy

All ssNMR analysis was performed on a high-field Bruker NMR AVANCE III spectrometer operating at 14 T (^1^H NMR frequency, 600 MHz) with a Bruker 2.5 mm MAS H–F/X double resonance probe. For ^1^H NMR, the signal was acquired at 25 kHz MAS by direct excitation with an rf field of 110 kHz. Interscan recovery time was set to 100 and 1.5 s for DTE and DTE@UiO-67 respectively; 16 scans were accumulated for each experiment. ^1^H–^13^C cross-polarization magic angle spinning (CP-MAS) excitation experiments have been acquired at the same spinning speed, Hartmann–Hahn conditions matched a ^13^C field of 70 kHz with a ^1^H field of 100 kHz (90–100 ramp) during a 9.5 ms contact time and ^1^H SPINAL decoupling. The interscan delay was set to 15 and 64 scans for acquisition on DTE, to 1.5 s and 1k scans for acquisition on DTE@UiO-67 and empty UiO-67. For ^19^F NMR, the signal was acquired at 28 kHz MAS frequency by direct excitation with a rf field of 87 kHz without ^1^H decoupling. Interscan recovery time was set to 80 and 1.5 s for DTE and DTE@UiO-67, respectively; 4 and 32 scans were accumulated for each experiment, respectively. Characteristic T_1_ relaxation times were measured with a saturation recovery pulse sequence for 10 different time intervals chosen to best describe the relaxation recovery curve. The ^19^F chemical shifts have been referenced indirectly with PTFE (−122.7 ppm) with respect to CCl_3_F.^68^ Cooling of the MAS sample was performed using a Bruker BCU-Xtreme cooling unit. Sample temperature was monitored by using a stator output thermocouple in the Bruker thermal control system. The real sample temperature was calibrated externally with PbNO_3_. The DMFIT program #20230120 ^69^ was used for spectral deconvolution and for fitting of CSA.

### Computational Details of NMR Calculations

Geometry optimizations and NMR calculations were carried out with the DFT-based CASTEP19.11 code^70,71^ using the Perdew–Burke–Ernzerhof (PBE) generalized gradient approximation (GGA).^72^ The structures were described as extended solids by using periodic boundary conditions. The wave functions were expanded using a planewave (PW) basis set with a kinetic energy cutoff and Brillouin zone *k*-point spacing chosen to produce converged results. Prior to NMR calculation, geometry optimization of all atoms was performed based on previously published X-ray structure.^73^ For the calculation of the isolated molecule, a molecule was taken from the unit cell and allowed to relax inside a cube of 30 × 30 × 30 Å. The so-called “ultra-soft” pseudopotentials (USPPs)^74^ were used to describe the interaction of the valence electrons with the nuclei and core electrons. The C19 “on the fly” pseudopotentials were used. The all-electron information was reconstructed using the GIPAW^71^ method. The wave functions were expanded using a PW basis set with a kinetic energy cutoff of 900 eV and integration over the Brillouin zone with *k*-point spacing <0.04 Å^–1^ on the Monkhorst–Pack (MP) mesh.^75^ The sizes of the standard and the fine grid were set to 2. The self-consistent field calculations were converged when the total energy of the system was stable within 10^–10^ eV. Geometry optimization was performed with semiempirical dispersion correction using the Tkatchenko–Scheffler scheme.^76^ The geometry optimization was performed with strict convergence tolerance criteria (1 × 10^–7^ eV/atom total energy convergence; 5 × 10^–3^ eV/Å max ionic force; and 5 × 10^–4^ Å max ionic displacement). NMR calculations were performed with the same pseudopotentials and convergence criteria as the geometry optimization and by including additional ZORA relativistic treatment.^77^

### Diffuse UV/Vis Reflection Spectroscopy (DRS)

Diffuse reflection spectra of all samples were collected by utilizing an Agilent Cary 5000 UV–vis–NIR Spectrophotometer. Measurements were performed in the range of 200 to 800 nm, before and after irradiation: Details on the irradiation processes can be found at the respective DRS spectra. All measurements were carried out at RT. For irradiation experiments, a Prizmatix PRI FC5-LED-WL (five high-power Fiberglas-coupled LED output with a potentiometer for manual power control) was used.

### IR Spectroscopy

IR spectroscopic measurements were performed on a Nicolet7500 FT-IR-Spectrometer under reduced pressure to avoid any contact with the humidity. For this, the sample and KBr were mixed in a 1:10 ratio and pressed to a thin transparent KBr pellet with a set pressure of ∼10 tons for 30 min. Scans were done in the range 360 to 4000 cm^–1^ with a resolution of 2 cm^–1^ and 90 scans per sample. The background (a pure KBr pellet) was measured with the same instrument settings as the sample and subtracted subsequently after each measurement. Data were collected before and after irradiation, the respective irradiation protocol is given at the respective IR spectrum. For irradiation experiments, a Prizmatix PRI FC5-LED-WL (five high-power Fiberglas-coupled LED output with a potentiometer for manual power control) was used. All measurements were performed at RT. The respective spectra of DTE, UiO-67, as well as DTE@UiO-67 can be found in Figures S4 and S5 and Supporting Information.

### Computational Details of DFTB3 MD Simulations

The two photoisomeric forms of DTE have been subjected to a conformational sampling at the GFN2-xTB^66^ level of theory using the CREST-3.0.2 software package.^78–80^ During the present study, exclusively the energetically most stable conformers of the open and closed forms have been used for the construction of the DTE@UiO-67 hybrid systems; see Figure 6, top.

The DFTB3 method,^40^ as implemented in the DFTB+ software suite version 24.1,^81^ has been used to carry out the quantum chemical force and energy calculations. The 3OB parameter set^82–85^ has been employed along with the DFT-D3 dispersion correction scheme according to Grimme et al.^86^ To accurately treat short-ranged interactions involving hydrogen, an exponent of 4.0 inside the damping function has been applied. Given the substantial size of the UiO-67 unit cell, Γ-point sampling of the Brillouin zone has been deemed adequate for modeling the periodic interactions. The convergence threshold for the self-consistent charge (SCC) cycles has been set to 10^–4^, ensuring that the convergence in energy is smaller than 10^–6^ Hartree.

MD simulations have been carried out utilizing the PQ-0.4.5 software.^77^ For time integration, the velocity Verlet propagator^87^ has been selected. The Bussi–Donadio–Parrinello thermostat^88^ together with the Berendsen manostat^89^ has been employed to achieve a sampling of the isothermal–isobaric ensemble at a temperature of 298.15 K and a pressure of 1.013 bar. All systems have been treated under isotropic conditions in pressure coupling. To allow for an increased time step of 2.0 fs, all hydrogen-involving bonds have been constrained to their average distance using the RATTLE algorithm.^90^

The initial structures of the DTE-closed@UiO-67 and DTE-open@UiO-67 hybrid systems have been constructed with the program PQAnalysis-1.2,^77^ by placing the corresponding photoisomer randomly into a large pore of the equilibrated host matrix while maintaining a minimum host–guest distance of 2.0 Å. Both hybrid systems have been re-equilibrated under *NVT* and *NPT* conditions before being sampled for a total of 1 ns. DTE@UiO-67 interaction energies *U*_int_ have been calculated according to the following equation.

$$U_{\mathrm{int}}=U_{DTE@UiO‐67}-\left\langle U_{UiO‐67}\right\rangle -\left\langle U_{DTE}\right\rangle$$

*U*_DTE@UiO‑67_, *U*_UiO‑67_, and *U*_DTE_ give the instantaneous total energy of the DTE@UiO-67 hybrid system, the pristine UiO-67, and the isolated DTE molecule, respectively. Angle brackets denote their time-averaged quantities.

## Results and Discussion

Synthesis of DTE@UiO-67 was performed via a gas-phase loading process to exclude any potential solvent molecule from all further considerations. This method has proven to be the most suitable synthesis protocol for the formation of dye@MOF systems to exclusively study the host–guest interactions e.g., in thioindigo@MOF^41^ or spiropyran@MOF^42,43^ systems.

The embedment process was followed by PXRD measurements, where modulations in reflection intensities compared to the nonloaded MOF were taken as evidence for the successful insertion. The respective PXRD patterns compared to the non-loaded UiO-67 can be found in Figure 2. Here, the modulations become especially apparent for the reflection at 4.26° 2θ, which experiences a significant increase in intensity for the hybrid system. To further follow the embedment, Rietveld refinement on the DTE@UiO-67 data was performed utilizing Topas.^44,45^ The respective plot as well as a comparison of the cell volume and lattice parameters with nonloaded UiO-67 can be found in Figure S1 and Table S1, Supporting Information. For DTE@UiO-67, a significant increase of the peak at 4.26° 2θ is found compared to nonloaded UiO-67 (see fit and difference curve, Figure S1, Supporting Information), which confirms the successful embedment of the photochromic dye. Notably, noninserted amorphous DTE on the MOF surface cannot be traced utilizing PXRD. Via liquid-state NMR spectroscopy, a composition of 0.9:1 DTE:UiO-67 was determined (see Figure S2 to S4 and Table S2, Supporting Information). Details of the calculation process can be found in Supporting Information.

**Figure 2:**
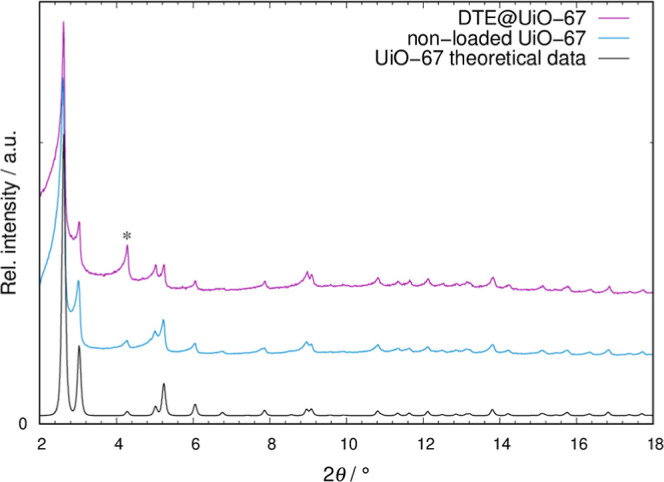
PXRD pattern of DTE@UiO-67 (pink line) in comparison to non-loaded UiO-67 (blue line) and theoretical data of UiO-67 (black line). An offset along *y* was applied for clarity. The asterisk marks the reflection with the most significant intensity change.

Within the IR spectrum of the nonirradiated system, all individual signatures (DTE and UiO-67) remain present, which validates the integrity of both components and with that the suitability of the synthesis protocol (see Figure S5, Supporting Information).

To further understand the impact of the individual components on each other, the effect of loading on both the MOF and the DTE was studied in detail by a combination of ssNMR measurements and density functional theory (DFT) calculations. As evidenced by Figure 3a, the ^13^C NMR spectrum of the pure MOF shows characteristic signals of the crystalline material.^46,47^

**Figure 3:**
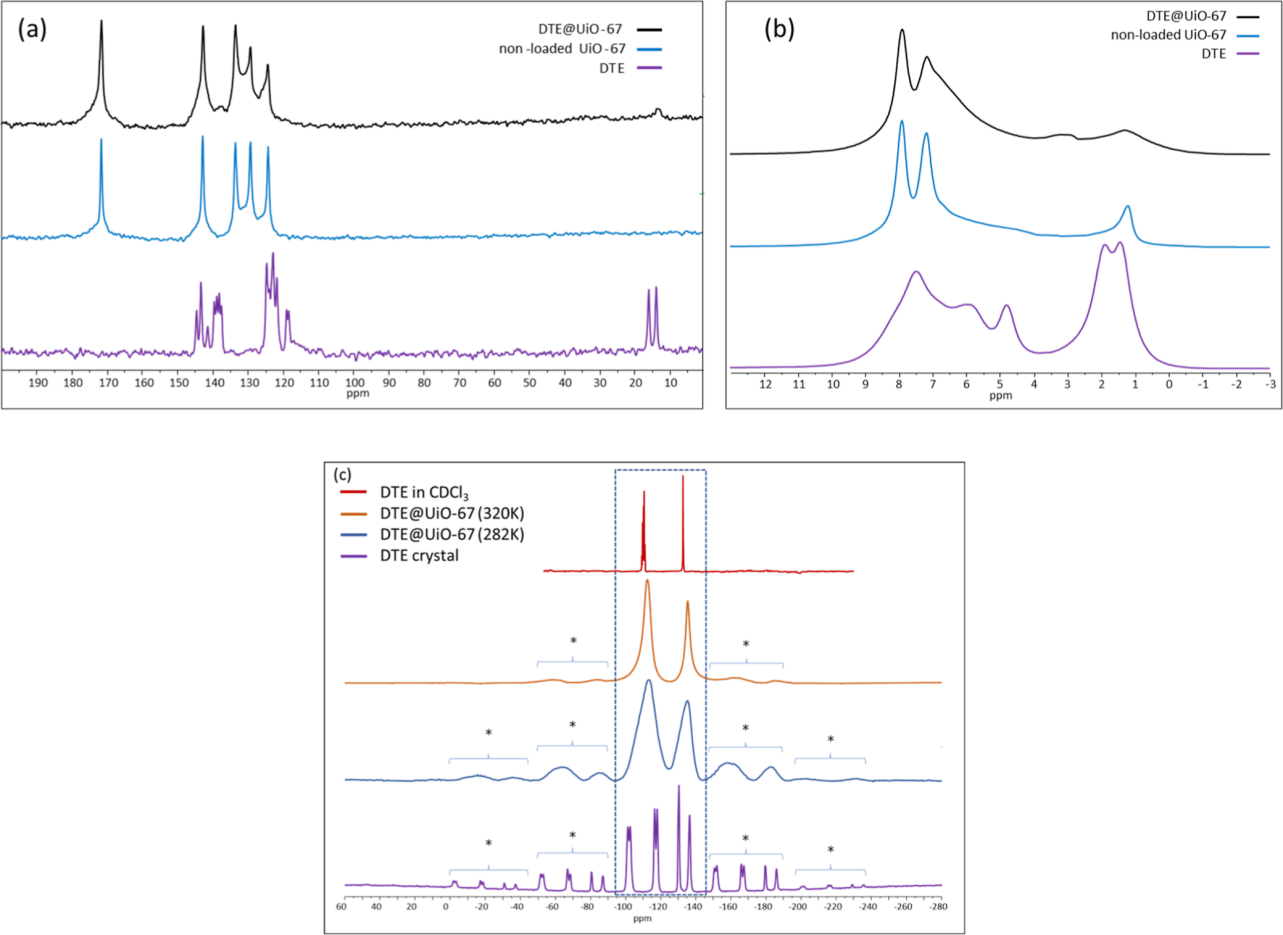
Comparison of solid-state ^13^C CP MAS NMR (a) and ^1^H MAS NMR (b) spectra of the pristine DTE (lower purple spectrum), nonloaded UiO-67 (middle blue spectrum), and DTE@UiO-67 system (top black spectrum). ^13^C CPMAS spectra were acquired for a “long” contact time (*ct* = 9.5 ms), which provided the most intense DTE guest signals inside the DTE@UiO-67 system; (c) comparison of solid-state ^19^F MAS NMR spectra of the crystalline DTE (lower purple spectrum), DTE@UiO-67 for *T* = 282 K (blue spectrum), and DTE@UiO-67 for *T* = 320 K (orange spectrum), with the solution NMR spectrum of DTE dissolved in CDCl_3_ (top-red spectrum). The central isotropic signals are highlighted by the dashed rectangle and the repetition of MAS sidebands is marked by the asterisks and the brackets. Solid-state spectra are acquired with a MAS sample spinning frequency of 28 kHz.

After DTE loading, these signals persist without resonance shifts, confirming that the host’s overall crystalline structure is retained. The slight broadening at the base of the MOF signals suggests local structural disorder within the pores induced by embedded DTE. In Figure 3b, ^1^H NMR spectra of the pure MOF and the hybrid system are compared. Consistently with ^13^C NMR, the ^1^H main resonances of the nonloaded MOF show typical chemical shifts at 7.9 and 7.2 ppm from the MOF linkers and the bridging Zr–OH hydroxyl protons from the hexameric unit (2–1 ppm). Additionally, some underlying broad components can also be distinguished, around 5 ppm corresponding to adsorbed H_2_O molecules, and around 2 ppm corresponding to residual solvent molecules and terminal Zr–OH hydroxyl protons interacting with H_2_O within the porous framework.^46–49^ Upon DTE loading, the overall spectrum significantly broadens. This is also consistent with local structural disorder induced by the guest and causes overlap with guest ^1^H resonances.

By focusing on the DTE NMR signature, ^1^H and ^13^C NMR of the pure DTE show characteristic resonances of the crystalline structure which are well resolved in the ^13^C spectrum (see Figure 3a,b). While the MOF Zr–OH hydroxyl protons prevent to distinguish the guest resonances from the host on the ^1^H NMR spectra, the DTE resonances around 138 and 13 ppm can be clearly identified on the ^13^C CPMAS spectrum of the hybrid system. In particular, it is striking that the two inequivalent methyl groups in the pristine crystal are no longer inequivalent within the MOF since only a single broadened signal is detected at 13 ppm. This indicates that the structure of the confined DTE is different inside the MOF. Since ^19^F nuclei are only present in DTE, their high NMR sensitivity makes them ideal spies to study the effect of confinement on DTE within UiO-67. Figure 3c compares the ^19^F spectra of the pristine DTE and the hybrid system at variable temperatures (282 and 320 K).

The isotropic central band shows that all six fluorine sites are well resolved for the pure DTE crystals, while this signature is not observed in the host/guest system. This indicates that no DTE crystallized outside the pores on the material surface and that the confined dye did not crystallize inside the system. On the other hand, only two relatively broad sites (at −113 and −135 ppm) with a respective integral ratio of 4:2 are observed in the DTE@UiO-67 system. This ratio and the corresponding chemical shifts match well with what is found in solution^50^ and the increased line widths point toward structural disorder that can have either static or motional origin. Additionally, the Magic Angle Spinning (MAS) sidebands (marked by asterisks in the figure) are significantly less intense when DTE is located inside UiO-67. This indicates a reduction of ^19^F chemical shift anisotropy (CSA) as would be obtained from partial averaging by rotational motion of the guest on a submicrosecond time scale. Since CSA of nuclei involved in polarized bonds in aromatic or rigid molecules is often dictated by intramolecular interactions, we verified this hypothesis by modeling NMR parameters by density functional theory (DFT) calculations.^51,52^ This allowed us to compare ^19^F CSA parameters for an isolated DTE molecule “in a box” with those of the same molecule structured inside the crystal. Indeed, as can be seen in Table 2, ^19^F Δ_CSA_ differences induced by the crystalline environment are quite negligible compared with the reduction of 10–25 ppm observed in the confined pore environment.

**Table 2:** Summary of NMR Parameters for the Different ^19^F Sites in DTE^a^

		^19^F sites	F1	F2	F5	F6	F3	F4
DFT calculations on DTE	crystal	Δ_CSA_ (ppm)	92.9	102.3	98.2	110.4	90	89.7
		ηCSA	0.4	0.5	0.4	0.1	0.3	0.4
	molecule	Δ_CSA_ (ppm)	94.6	101.6	94.3	113.7	93.9	88.3
		ηCSA	0.3	0.1	0.4	0.1	0.2	0.4
experimental NMR measurements	DTE crystal *T* = 320K	Δ_CSA_ (ppm)	90.3	94.4	88.5	96.9	79.2	83.1
		ηCSA	0.7	0.6	0.5	0.4	0.6	0.6
		fast rotating fraction	0%				0%	
		*T*_1_ (s)	60	62	60	62	60	62
	DTE@UiO-67 *T* = 282 K	Δ_CSA_ (ppm)	78.1				74.4	
		ηCSA	0.6				0.2	
		fast rotating fraction	10%				11%	
		*T*_1_ (ms)	377				337	
	DTE@UiO-67 *T* = 320 K	Δ_CSA_ (ppm)	70				57.7	
		ηCSA	0.5				0.6	
		fast rotating fraction	28%				27%	
		*T*_1_ (ms)	517				476	

a The table compares data obtained from DFT calculations and fitting of experimental data on pristine DTE and DTE@UiO-67 at variable temperature (more information on the NMR line width can be found in Table S2 and assignment of the ^19^F sites in the molecule can be seen in Figure S6).

The signal line width analysis reveals that MAS sidebands are about 2.5 times broader than the central isotropic resonance, indicating each central band consists of a fully isotropic narrow component (with no MAS sidebands) overlapped with a broader anisotropic signal (see Figure S6 and Table S2). This shows that the guest is not fully in a “liquid-like” state and that the spectra have to be fitted by a two-component model, accounting for fast-rotating ″liquid-like″ guests (with completely isotropic dynamics) and slower, more constrained guests, with only partial motional averaging of CSA. The significant effect of temperature on the ^19^F MAS NMR spectra of the confined DTE can be seen in Figure 3c, and Table 2 summarizes these effects by providing the evolution of CSA parameters and the proportion of “liquid-like” DTE at different temperatures. It can therefore be seen that by lowering the sample temperature from 320 to 282 K, the fast-rotating fraction of guest molecules is reduced from around 28% to 10%, and that the dynamics of the more constrained fraction, while being reduced or slowed down, are still averaging the ^19^F CSA to some extent. The table also compares *T*_1_ relaxation times, showing a sharp drop from *T*_1_ ≈ 60 s in the crystal to *T*_1_ ≈ 0.4 s in the MOF. This is also consistent with the presence of molecular mobility since *T*_1_ relaxation in solid-state NMR is often enhanced by molecular motion.^53^ Therefore, by carefully evaluating the characteristic ssNMR signatures of both DTE and UiO-67, DTE inclusion into the MOF pores can be confirmed, under the form of two guest fractions: An amorphous one with partial motional averaging of anisotropic NMR interactions and a “liquid-like” fraction with submicrosecond isotropic rotational dynamics.

In the next step, the optical response of DTE_0.9_@UiO-67 was studied. Upon irradiation with UV light, the open colorless DTE form converts to its closed colored DTE form; the switching process is depicted in Figure 1a. An elegant way to follow and quantify this photochromic reaction is to utilize IR^24,54–56^ or liquid-state NMR spectroscopy.^32,57^ The suitability of the first method has been shown for a diarylethene-based surface-mounted MOF (SURMOF),^58^ where the photochromic moiety is part of the linker backbone with a defined position. Here, the modulations of the bands between 700 and 850 cm^–1^ (vibrational changes of the methyl groups of diarylethene) served as probes for the determination of the photostationary state (PSS). For DTE within UiO-67 as a noncovalently attached guest, however, no changes in the IR signature are found, although the sample changed its color to pink (see Figure S7, Supporting Information). This could either be the result of the low density of DTE molecules compared to the aforementioned study^58^ or that the DTE molecule is preferably present in its photoinactive p form: the open-ring form of DTE occurs in a parallel (p) and in an antiparallel (ap) form (p and ap refer to the position of the two methyl groups) and only the antiparallel conformer is photoactive.^59,60^ In Figure 6c, both conformers are depicted. The amount of the photoactive *ap* conformer is dependent on the surrounding medium.^61,62^ In a previous work^32^ on DTE within MOF-5,^5^ MIL-68­(In),^63^ and MIL-68­(Ga),^63^ changes in the liquid-state NMR spectra^64,65^ before and after irradiation were used to study the photochromic reaction. Within those MOFs, only little DTE was present in the ap conformation, which resulted in small signals for the closed form of DTE. For DTE@UiO-67, The same irradiation experiments were performed. Although the solution turned pink, there is no signal appearing after UV-light exposure (see Figure S8, Supporting Information). Within the UiO-67 framework, the photoinactive p isomer of DTE seems to be preferred. Therefore, only a minor fraction is capable of being switched, and this fraction is responsible for the color change, which was traced by means of diffuse UV/vis reflectance spectroscopy (DRS), see following Figure 4.

**Figure 4:**
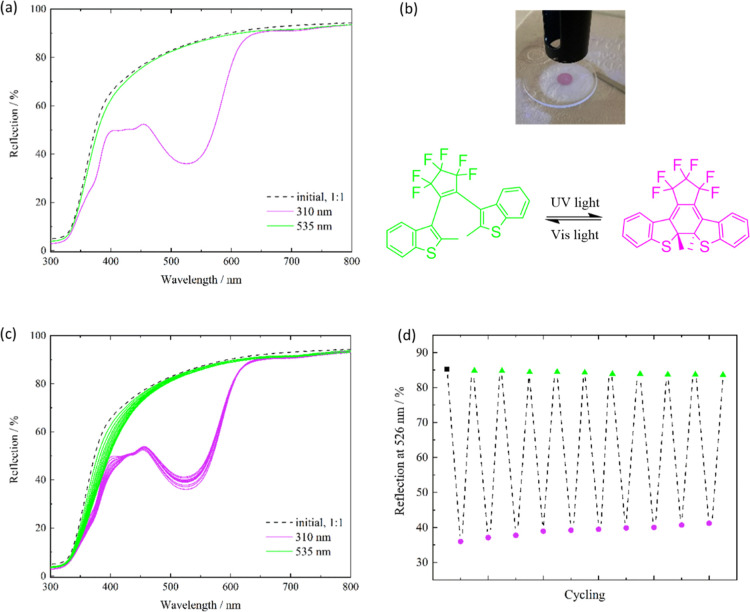
(a) DRS spectra of DTE_0.9_@UiO-67 before irradiation (black dashed line) and after irradiation with UV (λ = 310 nm, purple line) and visible (λ = 535 nm, green line) light, 5 min, respectively; (b) top: photograph of the irradiated sample; (b) bottom: visualization of the switching process with corresponding colors to the DRS spectra; (c) DRS spectra of DTE_0.9_@UiO-67 before (dashed black line) and after irradiation with 310 nm (purple line) and 535 nm (green line); (d) reflection of DTE_0.9_@UiO-67 at the closed DTE-specific reflection minimum wavelength (λ = 526 nm). For each irradiation wavelength, 10 switching cycles with an exposure time of 5 min were performed.

In the initial state (Figure 4a, dashed black line), no reflection minimum is present in the visible region, which corresponds to the open, colorless form of the DTE molecule. UV-light exposure results in a reflection minimum at 526 nm (Figure 4a, purple line) pointing to the formation of the closed DTE form (Figure 4b, top). The initial state is reached when the sample is irradiated with green light (Figure 4a, green line). Similarly to the previously reported switching properties of DTE within MOFs,^32^ photochromism can also be observed within UiO-67. Additionally, the fatigue resistance of the DTE_0.9_@UiO-67 composite material was tested. In Figure 4c,d, the results of the repetitive illumination are shown, which show a minimal fatigue within ten cycles of UV-light exposure. When embedded inside UiO-67, DTE shows only small hints of fatigue by solely focusing on the reflection minimum at 526 nm. The increase of this minimum after irradiation with λ = 310 nm is in total 14% after ten switching cycles, with 3% for the first, and ∼1% for the last. In comparison to the MOFs used in a previous study, UiO-67 is a chemically and thermally stable host, but the photoconversion of DTE is not beneficially influenced in terms of the ap conformer amount as well as switching performance. Notably, at around 400 nm, changes are found in comparison to the nonirradiated state. These changes in the UV region of the overall system are assumed to be the result of structural rearrangements during the isomerization process, which is also reflected in the switching performance from the open to the closed form.

To confront these findings on a structural basis, molecular dynamics (MD) simulations were conducted. Figure 5 illustrates the time evolution of the DTE@UiO-67 interaction energy. Examination of the final 0.5 ns of the MD simulations reveals that DTE exhibits interaction energies within UiO-67 of −32.6 and −36.5 kcal/mol, respectively, for the closed and open isomers.

**Figure 5:**
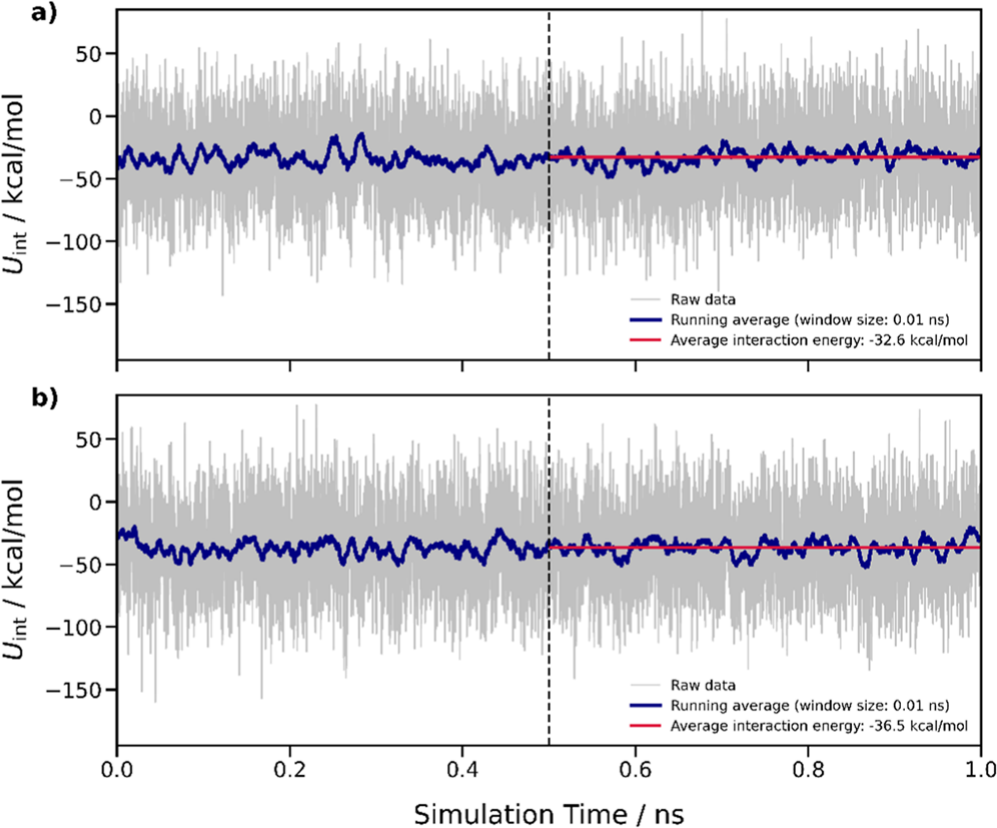
Time series of the DTE@UiO-67 interaction energies *U*_int_ for the closed (a) and open (b) form of the photoisomer. The blue lines indicate the associated running average over a window size of 0.01 ns. The dashed lines at 0.5 ns mark the starting point for determining the average *U*_int_ values of −32.6 and −36.5 kcal/mol, which are denoted by the red lines.

The stronger interaction of the open colorless DTE form can be rationalized by closer interaction with the host matrix. During the equilibration of the hybrid system, DTE in its open form transitions from a large into a small pore of UiO-67, while the closed form of DTE remains inside a large pore with its sulfur atoms oriented toward an inorganic node. Figure 6a visualizes the different pore occupations and binding motifs of the two forms of the photoswitch.

**Figure 6:**
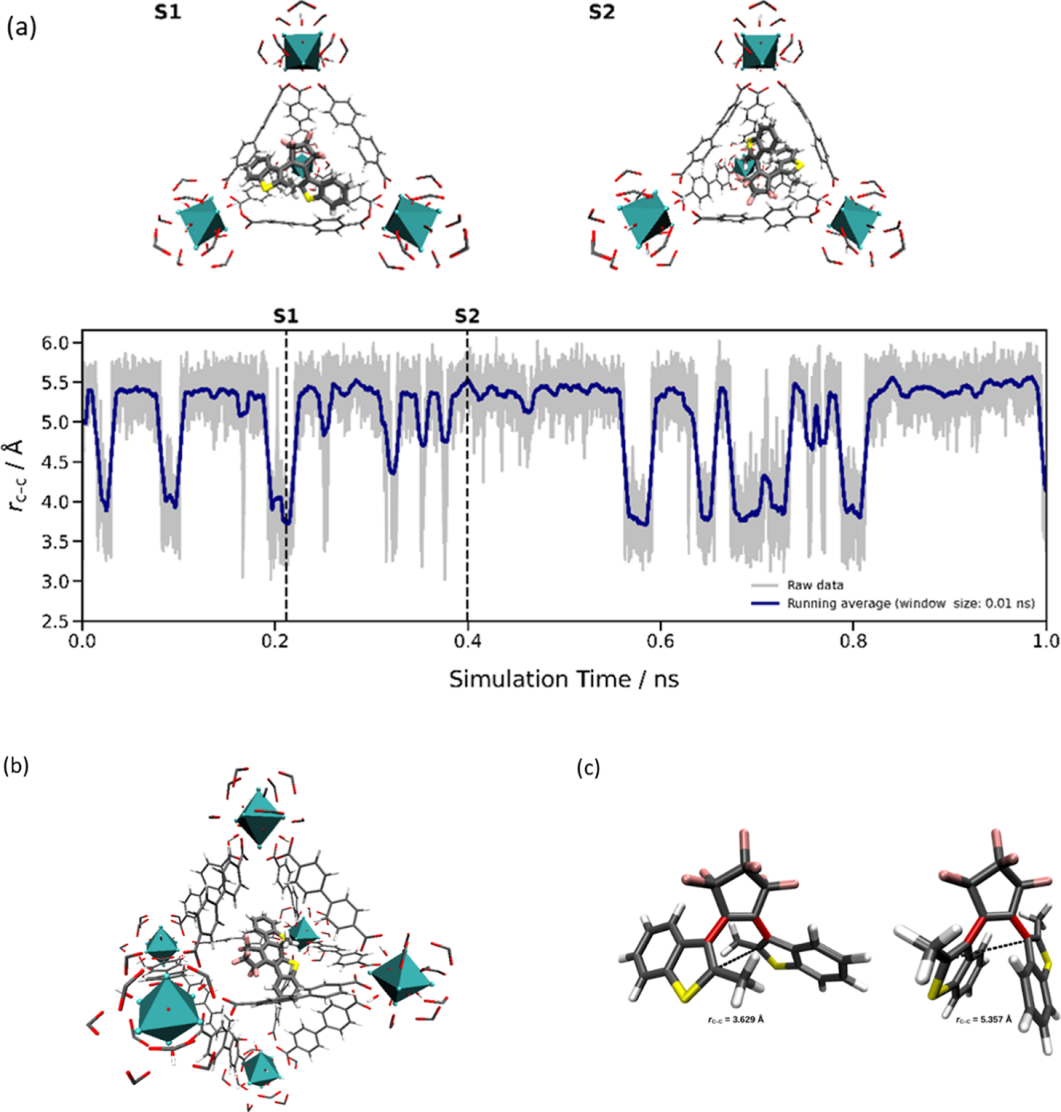
(a) Time evolution of the interatomic distance *r*_c–c_ of the two carbon atoms in DTE forming the bond during the photochemically induced electrocyclic reaction inside the UiO-67 host matrix. The guest dye moved into a small pore during the equilibration process and stayed there for the remaining trajectory. The two snapshots taken after 0.211 ns (S1) and after 0.399 ns (S2) show the open form of DTE in an unfolded conformation with a small *r*_c–c_ distance and in a more compact conformation with a larger carbon–carbon distance, respectively; (b) snapshot of the closed photoisomer of DTE inside a large pore of UiO-67 after 48 ps of sampling time. The guest dye started out in the same large pore and remained within this pore for the entirety of the simulation trajectory; (c) minimum configurations of the two conformers of the open form of DTE (p and ap conformers) obtained at the GFN2-xTB/GBSA­(CH3Cl) level of theory. Both conformers can interchange at standard conditions via internal rotation of the dihedral angle along the C–C bonds marked in red. Only the left conformer can be considered to be active in the photoisomerization process, since the large C–C distance of 5.357 Å between the associated carbon atoms (marked as dashed line) can expect to prevent the formation of a cyclic structure in case of the right conformer.

The interatomic distance of the two carbon atoms forming the bond during the photochemically induced electrocyclic reaction has been monitored for the DTE-open@UiO-67 system; see Figure 6a. A histogram showing the associated distributions is shown in Figure S9. The guest exhibits the capability to undergo a transition between two distinctly different conformations under the specified thermodynamic conditions. The minimum structures of the respective conformers of the open form DTE obtained at the GFN2-xTB level^66^ are shown in Figure 6c. Solvent effects in CHCl_3_ were treated using the generalized Born solvation model with solvent accessible surface area (GBSA).^67^ Both minimum configurations are accessible at standard conditions via rotation of the intramolecular dihedrals along the C–C bonds marked in red with a free-energy difference of only 0.17 kcal/mol in the case of the more compact conformer shown on the right in Figure 6c. Owing to the increased distance between the carbon atoms associated with the photoisomerization of 5.357 Å (highlighted by a dashed line), it can be concluded that only the left conformer enables the formation of the closed form of DTE. However, as seen in the MD simulation, after migration of DTE into the small pore of UiO-67, the compact form is preferred which provides a lucid rationale for the observed fatigue in the DRS measurements. As described, the interaction energies *U*_int_ for the closed and open forms with the MOF framework differ, making the open form the more favorable photoisomer within the MOF host. Both the migration of the dye as well as the stronger host–guest interactions are reflected in the optical response. Within MIL-68­(In), MIL-68­(Ga), and MOF-5, fatigue during several switching cycles has not been found for the DTE.^32^ The main difference between these studied systems and the present UiO-67 composite is the presence of only one pore (MOF-5 ^5^) or a channel-like structure (MIL-68­(In) and MIL-68­(Ga)).^63^

## Conclusion

Within this study, not only a new switchable hybrid material was presented but even more host- and guest-specific signature changes were carefully evaluated to understand the insertion process. The integration of DTE into the MOF pores was successfully confirmed through multiple techniques including powder X-ray diffraction and solid-state NMR spectroscopy. Further, utilizing ssNMR provided deeper insights into the structure and physical state of the dye inside the MOF scaffold. The study of the spectral line width and chemical shift anisotropy showed that the structural disorder induced by the dye is dynamic and that the guest is present in two different states with a ratio that depends on temperature. The optical behavior of DTE@UiO-67 was investigated by UV/vis diffuse reflectance spectroscopy, which demonstrated the typical photochromic response of DTE with a slight fatigue. Determination of the PSS was not possible due to the main presence of the open DTE in its *p* conformer and due to the migration of this open form of DTE to smaller tetrahedral pores within the MOF, as supported by molecular dynamics simulations. These simulations revealed that the interaction energies between DTE and the MOF are stronger in the open form of the dye, which favors its stability within the MOF. The simulations also indicated that the open form of DTE preferentially occupies the small tetrahedral pore, while the closed form remains in the large octahedral pore, leading to different interactions with the host material. Confrontation with the structural data obtained by ssNMR supports the hypothesis that the two fractions observed by NMR originate from guest molecules residing within pores of distinct sizes. In contrast to previously investigated MOFs, UiO-67 provides a unique nanoconfinement for the DTE molecule and unexpectedly not a gaseous or liquid-like environment to beneficially contribute to the photoswitching properties of DTE irrespective of its large pore size. This study demonstrates that each single MOF, and this is true for all porous materials, influences the optical properties of a noncovalently attached photoactive dye in a unique way. It is not only a matter of pore size, but also of, e.g., structure, polarity, or interaction forces. The freedom of the dye to “pick” its position, which impacts the overall material properties, makes it obligatory to establish a switch@MOF library for the systematic and precise evaluation of the influencing parameters.

## Supplementary Material



## Notes

Rietveld refinement of DTE@UiO-67; ^1^H NMR spectra of DTE, UiO-67, and DTE@UiO-67; IR spectra of pure DTE compared to UiO-67 as well as DTE@UiO-67; spectral deconvolution of DTE@UiO-67 variable-temperature ^19^F MAS NMR spectra; IR spectra of DTE@UiO-67 before and after irradiation; liquid-state IR spectra of DTE dissolved in toluene before and after irradiation; histogram showing the C–C distance in DTE; comparison of cell volume and lattice parameters of DTE@UiO-67 and pristine UiO-67; relevant values for the determination of composition of DTE@UiO-67 by liquid-state NMR; and relevant values obtained from fitting DTE@UiO-67 variable-temperature ^19^F MAS NMR spectra (PDF)

## §

Surname was formerly Schwartz.
