# Supplementary material for: Structure–Function Correlation in Switchable DTE@MOF Hybrids: Tracking Local Dynamics and Fatigue Pathways
Source: J Phys Chem C Nanomater Interfaces. 2025 Dec 2;129(50):22179–90. doi: 10.1021/acs.jpcc.5c06348 (PMC12720239; doi:10.1021/acs.jpcc.5c06348)
Supplement: Supplementary file 1 [file jp5c06348_si_001.pdf]

Supporting Information

Structure–Function Correlation in Switchable DTE@MOF Hybrids: Tracking Local Dynamics and Fatigue Pathways

Markus Rödl[a], Eva Neuner[a], Armin Penz[a], Axel Gansmüller[b], Thomas S. Hofer[a,*], and Heidi A. Reichl (formerly Schwartz)[a,*]

[a] *Institute of General, Inorganic and Theoretical Chemistry, Universität Innsbruck, Innrain 80-82, 6020 Innsbruck, Austria,*

[b] *CNRS, CRM2 UMR 7036, Université de Lorraine, Boulevard des Aiguillettes, BP 70239, 54506 Vandoeuvre les Nancy, France,*

Email corresponding author main: heidi.reichl@uibk.ac.at

Email corresponding author theory: t.hofer@uibk.ac.at

Content

Figure S1. Rietveld refinement on DTE@UiO-67.

Figures S2 to S4. ^{1}H NMR spectra of DTE, H_2BPDC, UiO-67 and DTE@UiO-67.

Figures S5. IR spectra of pure DTE compared to UiO-67 as well as DTE@UiO-67.

Figure S6. Spectral deconvolution of DTE@UiO-67 variable temperature ^{19}F MAS NMR spectra.

Figure S7. IR spectra of DTE@UiO-67 before and after irradiation.

Figure S8. ^{1}H NMR spectra DTE@UiO-67 before and after irradiation (λ = 310 nm).

Figure S9. Histogram showing the C-C distance in DTE.

Table S1. Comparison of the lattice parameters and cell volumes of DTE@UiO-67 to pristine UiO-67.

Table S2. Relevant values for the determination of composition of DTE@UiO-67 by liquid-state NMR.

Table S3. Relevant values obtained from fitting DTE@UiO-67 variable temperature ^{19}F MAS NMR spectra.

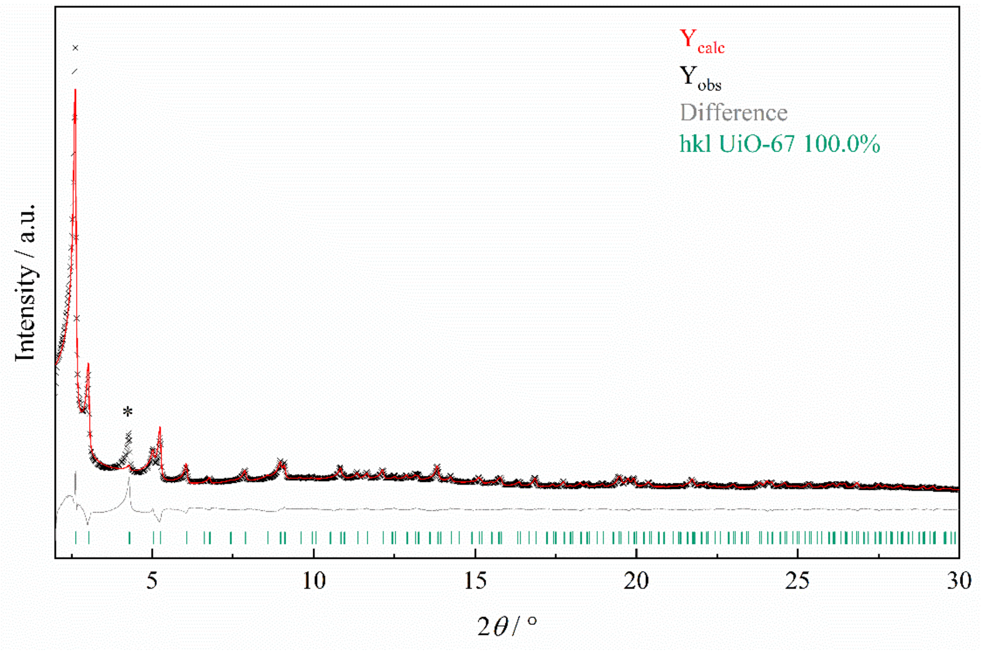

Figure S1. Diffraction pattern (black, 298 K/*Stoe Stadi P*: $\lambda = 0.7093$ Å) and Rietveld refinement (red line) of DTE@UiO-67. The reflection positions of UiO-67 are shown in green ($R_{exp} = 60.95\%$, $R_{wp} = 7.71\%$, $R_{p} = 5.01\%$, GooF = 8.12). The asterisk marks the most significant peak intensity modulation compared to the non-loaded MOF.

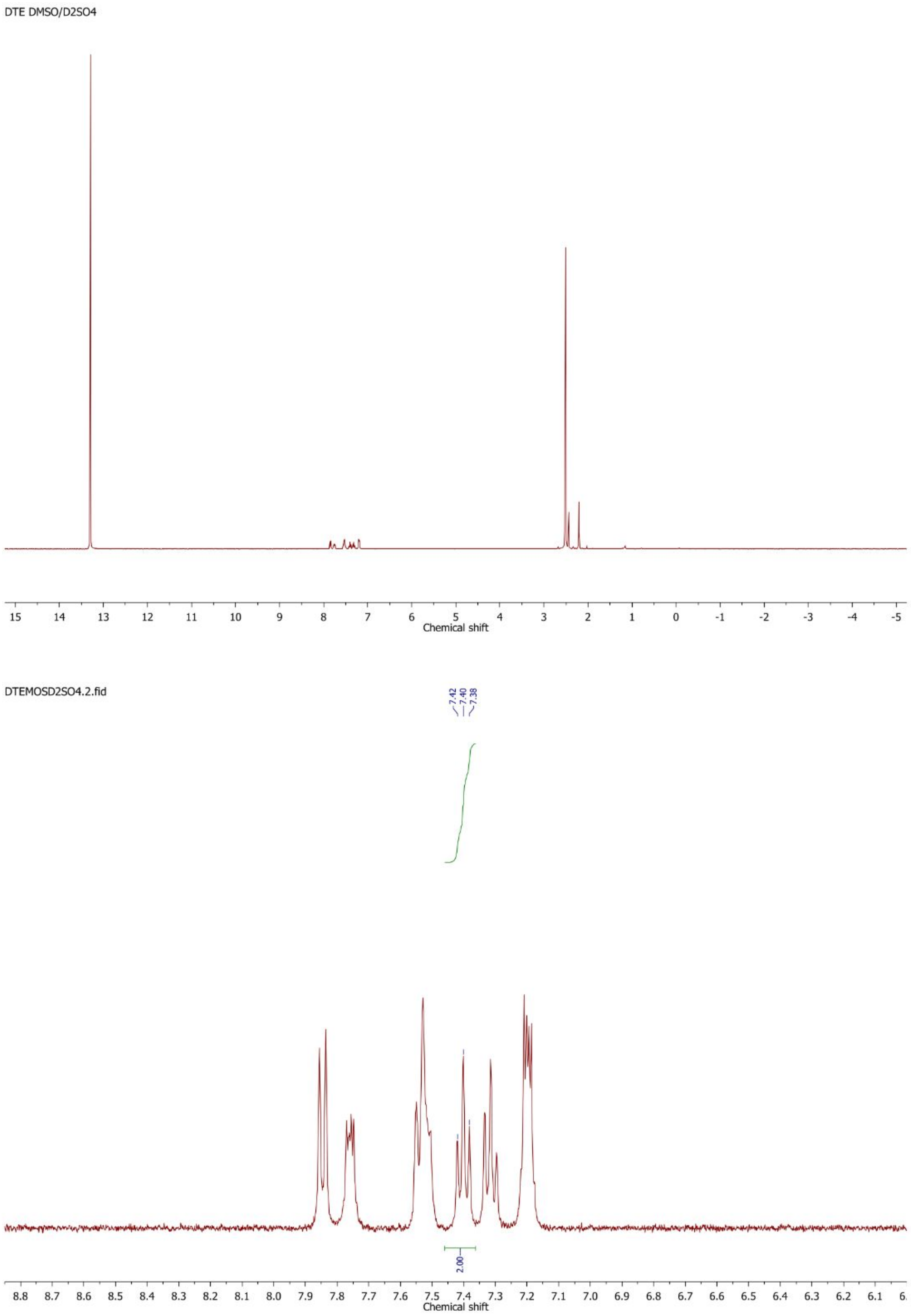

Figure S2. Top: ^{1}H NMR spectrum of DTE digested in DMSO-d_6 and D_2SO_4; bottom: zoom-in into the aromatic region. The signal at [illegible] 13 ppm corresponds to $HDSO_4$.

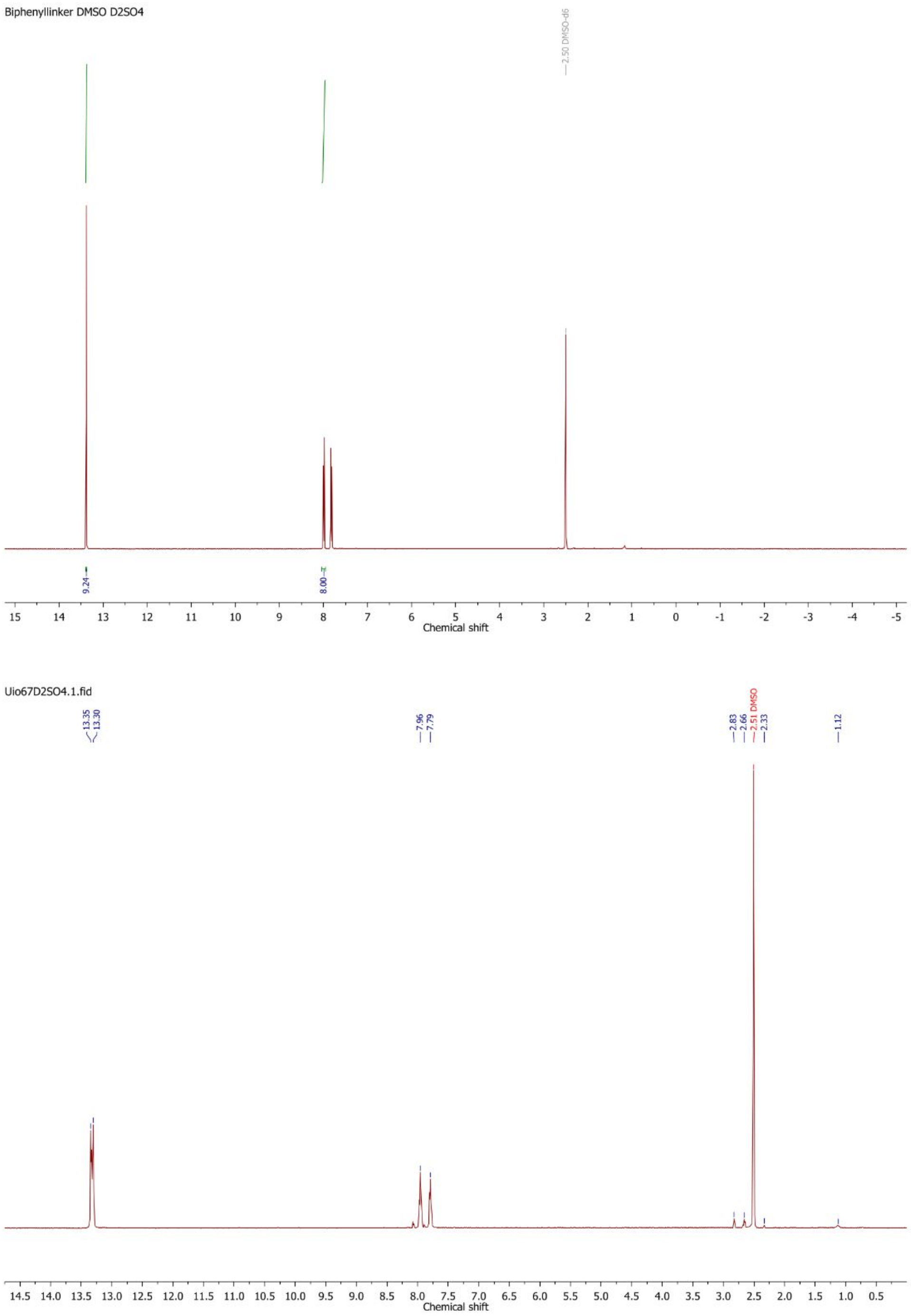

Figure S3. Top: ^{1}H NMR spectrum of biphenyl-4-4'-dicarboxylic acid (H_2BPDC) digested in DMSO-d_6 and D_2SO_4; bottom ^{1}H NMR spectrum of UiO-67 digested in DMSO-d_6 and D_2SO_4. The signal at □ 13 ppm corresponds to $HDSO_4$. The signals at □ 8 ppm correspond to the aromatic protons of the linker.

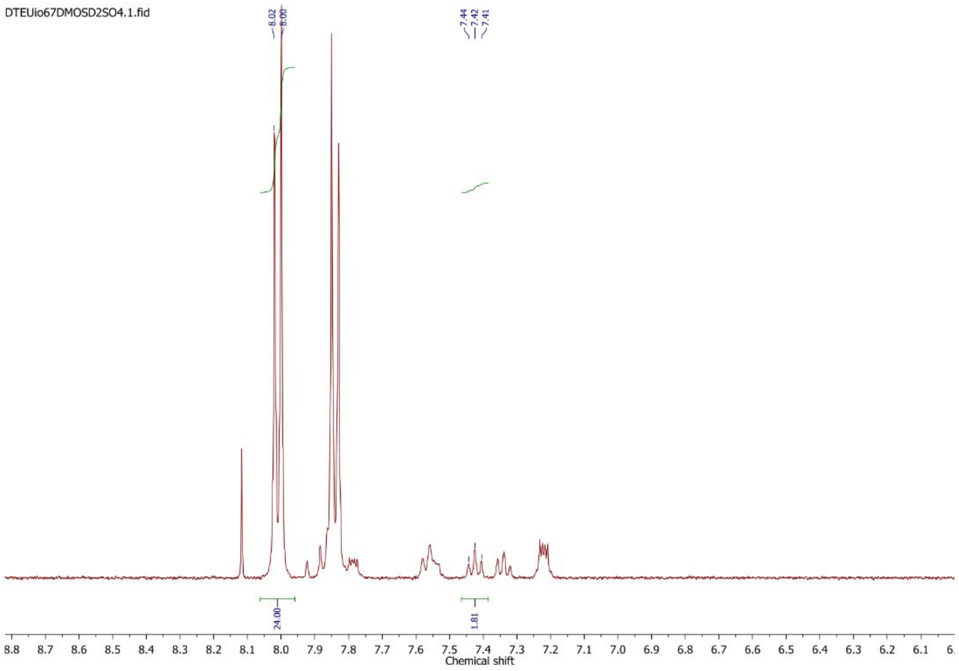

Figure S4. ^{1}H NMR spectrum of DTE@UiO-67 digested in DMSO-d_6 and D_2SO_4.

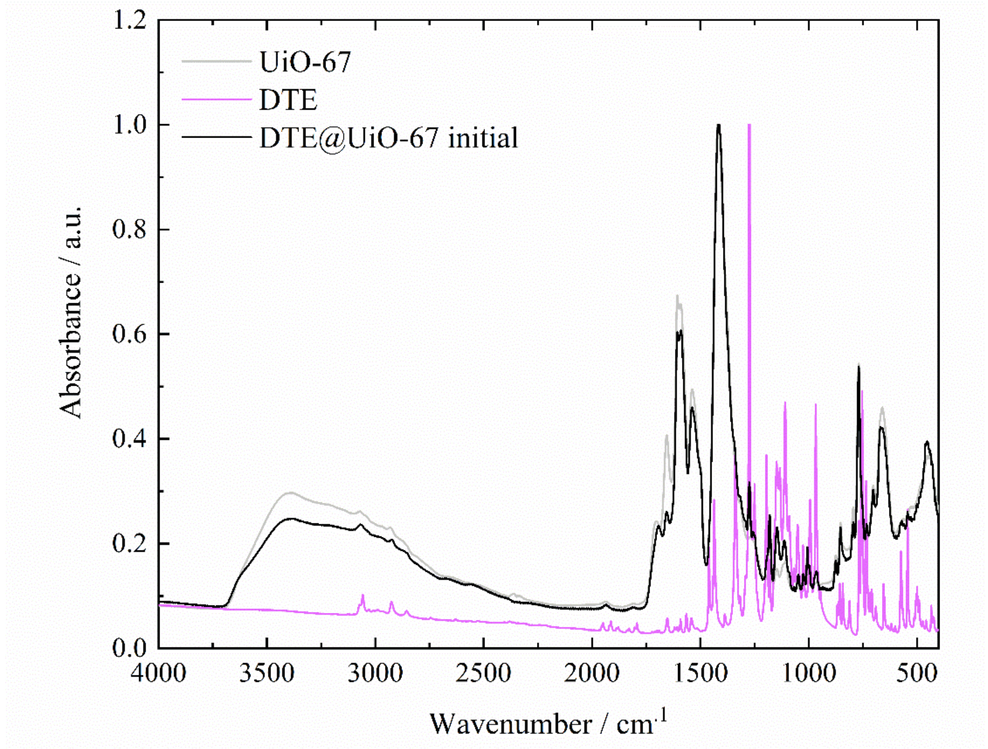

Figure S5. IR spectra of pure UiO-67 (grey line), pure DTE (pink line) and DTE@UiO-67 (black line).

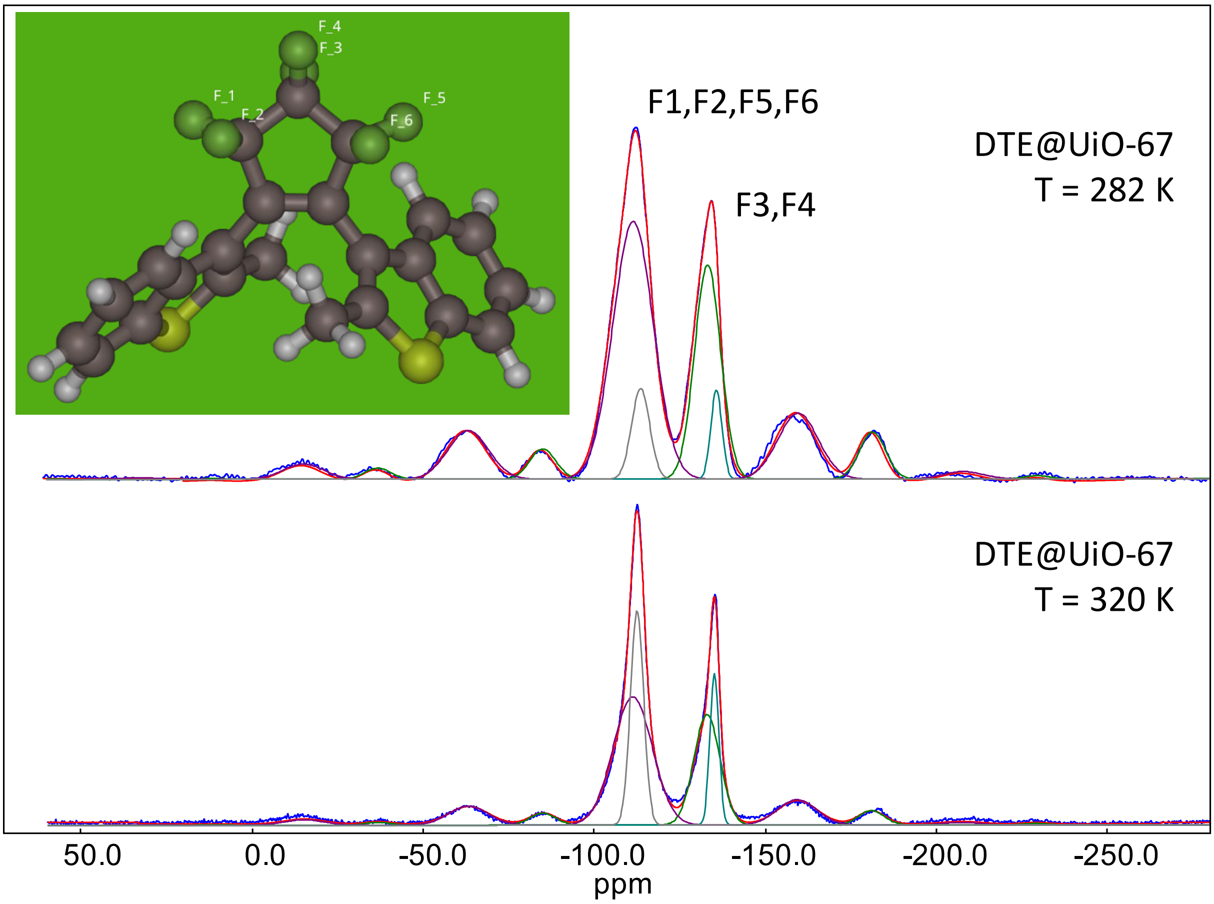

Figure S6. ^{19}F MAS NMR spectral deconvolution from DTE@UiO-67 at 282 K and 320 K. Deconvolution has been performed with the Dmfit software and the results are summarized in table S3. The inset on the top left presents the assignment of the ^{19}F sites inside the DTE molecule.

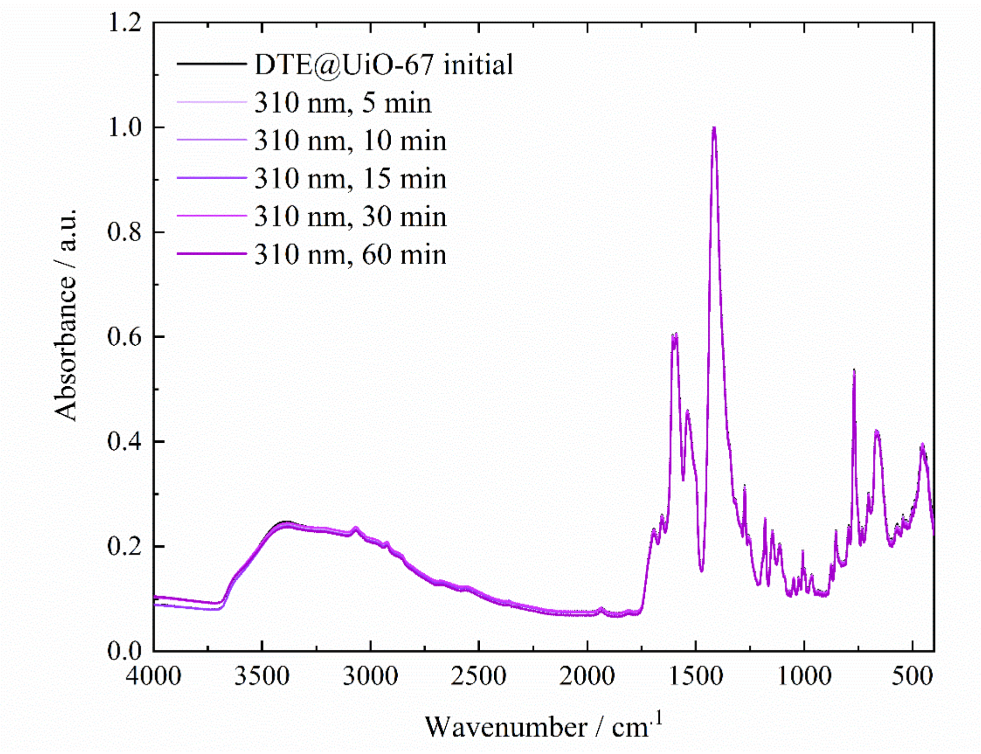

Figure S7. IR spectra of DTE@UiO-67 before (black line) and after irradiation with UV light (λ = 310 nm, pink lines).

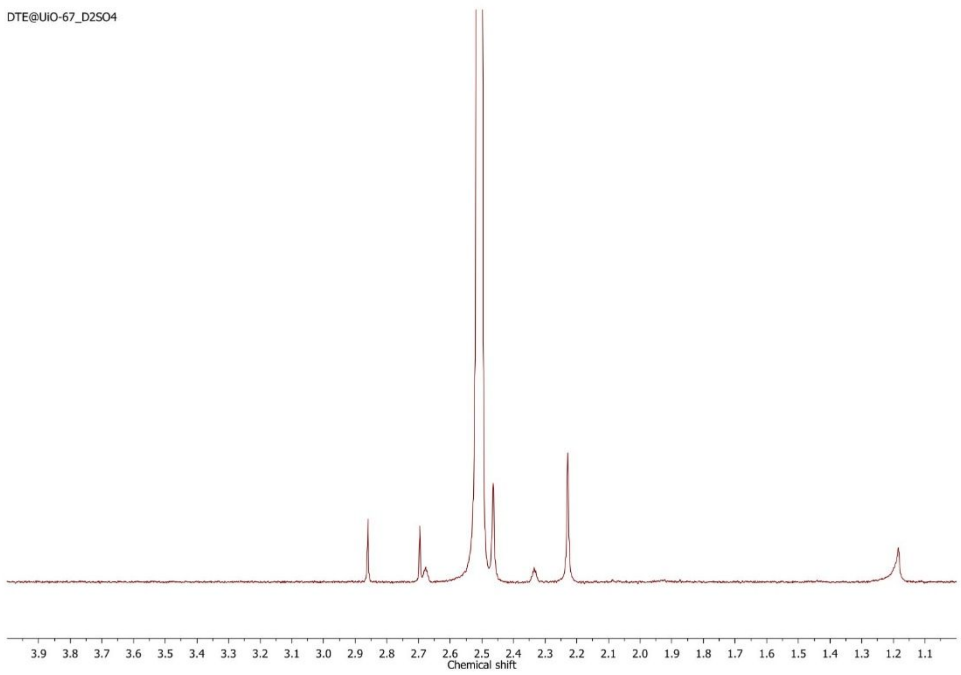

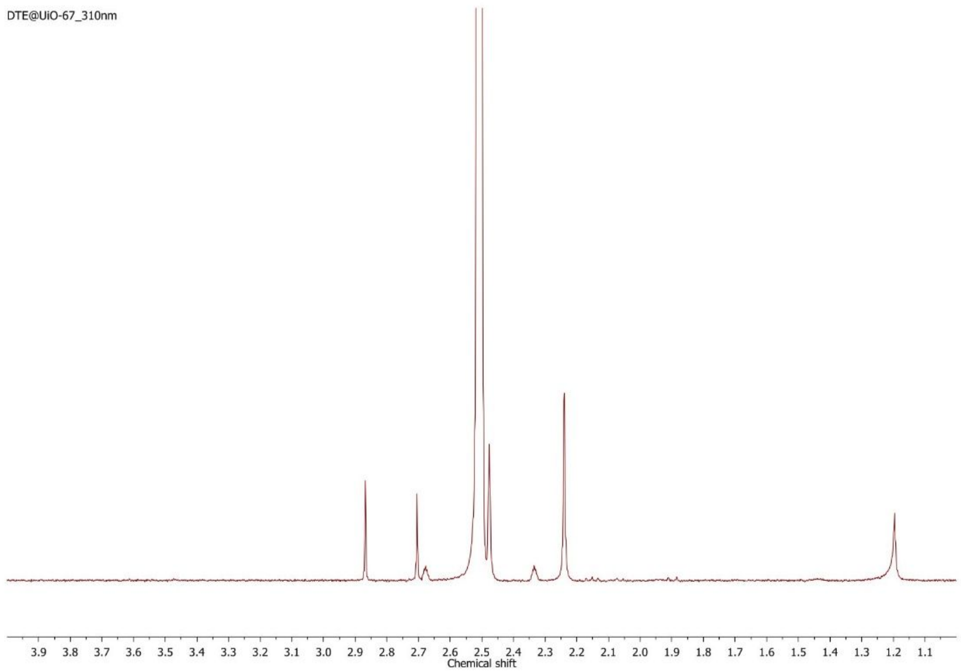

Figure S8. ^{1}H NMR spectrum of DTE@UiO-67 digested in DMSO-d_6 and D_2SO_4 before (top) and after irradiation with UV light (bottom).

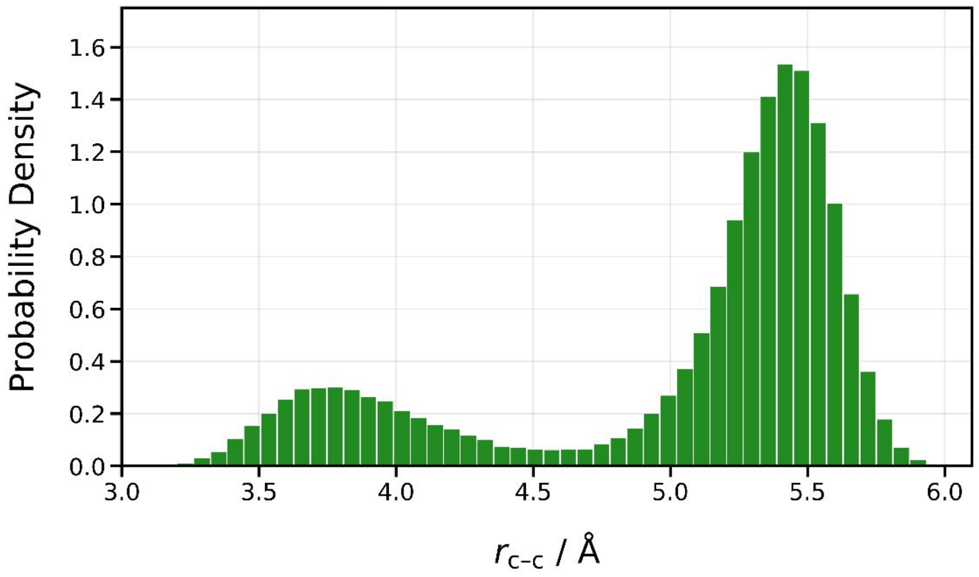

Figure S9. Distribution of the interatomic distance of the two carbon atoms in DTE forming the bond during the photochemically induced ring-closing reaction inside the UiO-67 host matrix during the 1 ns MD simulation trajectory of DTE@UiO-67. The histogram has been generated using a total of 50 bins. 22,1% of values are lower than 4.5 Å, while 77.9% are greater than 4.5 Å.

Table S1. Comparison of the lattice parameters and cell volumes of DTE@UiO-67 to pristine UiO-67.

	UiO-67[1]	DTE@UiO-67
Temperature / K	100 K	298 K
Space group	$Fm\bar{3}m$	$Fm\bar{3}m$
Lattice parameter a / Å	26.8809(3)	26.7928(82)
Cell volume / Å^3	19423.7(4)	19233(18)

Composition determination *via* NMR. NMR measurements were performed to determine the composition of DTE@UiO-67. For this, the characteristic proton signals of both the MOF and the guest were integrated and related to each other. In detail, the following signals were chosen, which are listed in the following Table S1.

Table S2. Relevant ^{1}H NMR signals of DTE as well as UiO-67 used for the determination of composition *via* NMR.

compound	composition	^{1}H signal / ppm	Number of protons
DTE	$C_{22}H_{15}F_9O_{10}S_3$	7.4 (t)	2
UiO-67	$Zr_6(C_{14}H_8O_4)_6$	8 (d)	24

In a next step, the integrals of these characteristic signals (see Figure S3) were related to each other by considering that 24 protons account for one formular unit MOF and 2 protons account for one formular unit DTE. The signal at 8 ppm was set to 24, which resulted in an integral of 1.81 at 7.4 ppm. Conclusively, the molar DTE-to-UiO-67 ratio is 0.9:1.

Table S3. Relevant ^{19}F MAS NMR spectral parameters obtained from the fitting for DTE@UiO-67 at T = 320 K and T = 282 K.

	^{19}F sites	F1, F2, F5, F6		F3, F4	
		amorphous	liquid like	amorphous	liquid like
DTE@UiO-67 T = 320 K	**fraction**	**72%**	**28%**	**73%**	**27%**
	δ_{iso} (ppm)	-111.2	-112.5	-132.9	-135.1
	Linewitdth (Hz)	7635	2558	5041	1720
	Δ_{CSA} (ppm)	70.0	0	57.7	0
	ηCSA	0.5	0	0.6	0
DTE@UiO-67 T = 282 K	**fraction**	**90%**	**10%**	**89%**	**11%**
	δ_{iso} (ppm)	-111.7	-113.9	-133.4	-136.0
	Linewitdth (Hz)	7815	3507	5030	2132
	Δ_{CSA} (ppm)	78.1	0	74.4	0
	ηCSA	0.6	0	0.2	0

References

(1) Øien, S.; Wragg, D.; Reinsch, H.; Svelle, S.; Bordiga, S.; Lamberti, C.; Lillerud, K. P. Detailed Structure Analysis of Atomic Positions and Defects in Zirconium Metal-Organic Frameworks. *Cryst. Growth Des.* **2014**, *14* (11), 5370–5372. https://doi.org/10.1021/cg501386j.
